# Supplementary material for: Are carbon emissions trading and green financial instruments synergistic? -Comprehensive quantitative research based on content analysis
Source: PLoS One. 2024 Mar 7;19(3):e0298601. doi: 10.1371/journal.pone.0298601 (PMC10919601; doi:10.1371/journal.pone.0298601)
Supplement: S1 Data — (ZIP) [file pone.0298601.s001.zip › Data/地级市Z_F工作报告.zip!/╣π╢1⁄2/╔╟═╖╩╨/└┤╫╘╣1⁄2╓┌║┼í╛┬φ┐╦╩2╛▌═°í┐_2019.docx]

各位代表：

现在，我代表汕头市人民政府，向大会作政府工作报告，请予审议，并请市政协委员和其他列席人员提出意见。

一、2018年工作回顾

过去一年，是改革开放40周年，是汕头改革开放进程中很不平凡的一年。3月7日，习近平总书记参加十三届全国人大一次会议广东代表团审议并发表重要讲话，对汕头发展寄予殷切希望。10月22日至25日，习近平总书记视察广东并发表重要讲话，明确要求加快汕头经济特区发展，把汕头作为重要发展极，打造现代化沿海经济带，赋予了汕头新的更高的战略定位和要求，为汕头新时代改革开放再出发提供了根本遵循、注入了强大动力。一年来，我们牢记习近平总书记的嘱托，以习近平新时代中国特色社会主义思想为指导，全面贯彻落实党的十九大和十九届二中、三中全会精神，在省委、省政府和市委的坚强领导下，在市人大及其常委会和市政协的监督支持下，坚持稳中求进工作总基调，落实高质量发展要求，主动应对外部环境深刻变化，凝心聚力、攻坚克难，经济社会保持平稳健康发展。预计全市实现地区生产总值2565亿元，增长7%；一般公共预算收入131.52亿元，可比增长6.6%；固定资产投资2082亿元，增长19%；社会消费品零售总额1772亿元，增长9%。

（一）实体经济稳中趋优。惠企暖企护企有新举措，全面落实中央、省、市支持实体经济发展系列政策措施，投入促进民营经济发展等专项资金12亿元，共降低制造业企业成本40亿元。实施市领导挂钩联系制度，扎实解决企业困难和问题。成立纾困基金，多渠道帮助上市企业纾解股权质押风险问题。加强企业培育，新增规上限上企业293家，新认定省高成长中小企业40家，新增“新三板”挂牌企业3家，在广东证监局备案和在证监会排队准备上市企业共13家。新兴产业有新发展，上海电气风电集团海上智能制造项目落地建设，比亚迪二期3D玻璃项目部分实现投产，中海信（汕头）创新产业城一期项目基本建成。实施新一轮技术改造，工业技改投资规模、增速分别列全省第三、第二位。产业集聚有新突破，市产业转移工业园再次获省考评“优秀等次”，获“广东省五星级服务园区”称号。促进产融合作，新设立融资租赁公司5家、私募基金3家，金融机构贷款余额增速列全省第一位。深化两化融合，入选“广东省工业互联网创新应用试点区”和“广东省超高清（4K）电视网络应用试点示范城市”。推动工业设计创意，举办第二届“市长杯”工业设计大赛，金平工业设计中心、龙湖工业设计城开园。质量工作获省政府考核最高等级A级。品牌兴市有新成效，省名牌产品达128个，澄海区获批创建“全国动漫产业（玩具）知名品牌示范区”，濠江区获批创建“全国滨海潮汕文化休闲旅游知名品牌示范区”。启动全域旅游示范市创建工作，旅游总收入增长20%。

（二）创新驱动成效突出。重大科研平台建设取得新突破，化学与精细化工广东省实验室正式挂牌运作，与9个院士团队及境内外22家高校、科研院所建立合作关系。加强产学研协同创新，汕头和广东工业大学共建的协同创新研究院、汕头机器人与智能制造研究院、“一带一路”科技创新与服务研究院挂牌运行。创新主体建设有明显进展，新增省级工程技术研究中心48家，光华科技国家企业技术中心获国家认定，广东省智能化超声成像技术创新中心获批筹建。443家企业通过科技型中小企业评价，新增国家级高新技术企业198家，总量达到718家，实现三年翻两番。设立总规模6亿元的粤科汕瑞创业投资基金，举办汕头创业之星大赛，一批创新孵化载体、平台、空间实现规模化发展。加大对基础与应用基础研究支持力度，首次设立国家、省自然基金项目培育计划和医学科技人才培育、临床科研技术提升计划，开设专项支持省级重点实验室和国有科研机构开展应用基础研究。R&D经费投入增长30.2%，列全省第二位。获批国家知识产权示范城市，发明专利申请量和授权量分别增长30%和4.5%，取得中国专利优秀奖和外观专利优秀奖11项、省专利优秀奖5项。新业态蓬勃发展，淘宝镇13个，淘宝村93个，分别列全省第一、第四位，潮阳、澄海区获评省级电子商务进农村综合示范区。

（三）改革开放全面深化。制定加快推进新时代全面深化改革的实施意见、美丽汕头发展战略规划（2018－2050），推动18项中长期重要改革工作。营商环境综合改革成效明显，成为全省4个试点城市之一。42个单位985个事项进驻网上办事大厅，办理时限缩短50%以上，办理速度全部达到全省前三，营商环境指标对照世界银行评价体系取得明显进步。深入推进“一门式一网式”政府服务模式改革，形成“综合窗口+专业窗口”服务模式，实现实体办事大厅“一站式服务”功能。深化商事制度改革，新登记企业数增长16.8%。推进国家社会信用体系建设示范城市创建工作，城市信用排名列全省地级市第三位。政府工作部门机构改革进入全面实施阶段。推动国有企业改革重组，组建市超声、投融资、建工、冷链物流、交通运输、资产管理等六大集团公司，优化国资布局和结构。推进农信社改制农商行工作，南澳农商行挂牌开业。推进农村改革，基本完成农村土地确权颁证和澄海区农村集体产权制度改革试点工作。加快构建全面开放新格局。汕头保税物流中心（B型）封关运营，成为粤东地区唯一入中心即可退税的海关特殊监管场所。作为省提升跨境贸易便利化水平试点地区，率先开展18项口岸业务改革。国际贸易“单一窗口”上线应用功能达20项，实现口岸业务流程无纸化，口岸通关效率居全国前列。积极应对中美贸易摩擦，深化与“一带一路”沿线国家经贸文化合作。推进中欧国际物流新通道发展，汕头—明斯克国际货运班列开通试运行。吸收外资大幅增长，合同外资增长362.8%。

（四）“四大平台”加快推进。华侨试验区改革创新力度加大，全国首创电信基础设施统一规划、共建共享模式，全国首创“助企贷”新模式，试点建设省海岸带综合示范区；人才大厦投入运营，泰盛科创园、明园科创金融城、潮商金融中心等加快建设；津湾公园、国家示范性高中开建。汕头国家高新区拓展至30平方公里，创新功能区和行政区新型管理体制，中以（汕头）科技创新合作区发展战略规划方案通过专家评审。自贸试验区申报工作扎实推进，对标广东自贸试验区385个项目改革创新经验和案例，复制推广154项自贸区改革创新经验。江湾新区启动高标准规划建设，发展总体规划通过专家评审。

（五）基础设施有效突破。掀起新一轮投资建设热潮，开展第四季度重大项目集中签约开工暨竣工投产活动，共签约、开工、投产重点项目211个，总投资额1016亿元，呈现投资领域广、投资规模大、产业层次高、惠及民生多等特点。拓宽重点项目建设资金渠道，新增地方政府债券资金47亿元，PPP项目投融资累计达649亿元。完成基础设施投资450亿元，增长30%。交通基础设施投资首次突破百亿大关。广澳港区西防波堤主体工程完工，10万吨级航道基本建成，二期工程两个10万吨级集装箱码头主体工程基本完成。广梅汕铁路增建二线及厦深联络线、汕头火车站综合枢纽首期工程建成运营，实现动车进城，汕汕高铁开工建设，疏港铁路工可、城市轨道交通建设规划加快报批。揭惠高速全线建成通车，汕湛高速、潮汕环线高速全面铺开建设。完成国省道改造110公里。汕头海湾隧道、牛田洋快速通道、中山路东延、凤东路等重大交通市政基础设施加快建设，海滨路东延一期工程动工建设，泰山北路建成通车。环保基础设施投入加大，总投资127亿元的练江流域（汕头段）11个污水处理厂及730.8公里配套管网全面启动建设，新溪污水处理厂建成运营，中心城区、澄海、潮阳、潮南区生活垃圾焚烧发电厂相继建成投入使用。水利、能源、信息化基础设施建设扎实推进，三江水系连通工程潮水溪疏浚工程启动建设，海门湾桥闸和练江水闸重建工程、一批电网工程、汕特燃机电厂天然气热电联产项目开工建设，洋东、勒门I、勒门II、海门等海上风电项目开展前期工作，新增4G基站1306座、物联网基站265座。

（六）城乡环境明显提升。持续推进创文提质升级。顺利通过“国家卫生城市”复审、“国家园林城市”省级复审。新改建市政道路26条159万平方米，新铺设步道80公里、雨污分流管道121公里，完成海滨路提速改造，实施黄河路、泰山路快速化改造，道路通达能力明显提升。升级改造农贸市场46个，新建改造公厕247座，升级改造公园、绿地30万平方米，在牛田洋片区建成11公里的全省最长红树林景观带，市容环境持续美化。小公园开埠区修复改造一期工程基本完成，二期工程开工建设；乌桥岛棚户区改造项目加快推进，已开工棚改房2000套，老城区焕发新光彩。乡村振兴取得新进展。建设美丽乡村示范村96个，澄海、潮南区实现村村通自来水，启动美丽宜居村建设238个，建成农村公路156公里、安保工程250公里，建制村通硬化路和客运班车率均达100%，农村人居环境明显改观。生态环境治理力度加大。全面落实中央环保督察组“回头看”整改要求，“2+2+10”环保基础设施加快建设，持续开展练江流域生活垃圾堆放点、畜禽养殖污染、“散乱污”企业整治、干支流“五清”、环境违法打击等五大专项行动，实现园区外印染企业全面依法关停，练江整治实现重大转折，练江干流各项污染物指标持续下降，沿岸环境持续改观。全面实行河长制湖长制，积极开展百河千沟万渠大整治，逐步实现城乡“堤固、河畅、水清、岸绿、景美”的整治目标。南澳“蓝色海湾”整治项目成为生态修复典型范例。环境空气质量综合指数排名列全省第四，县级以上集中式饮用水水源地水质100%达标，城市生活垃圾无害化处理率达100%。

（七）人民生活持续改善。坚持以人民为中心的发展思想，持续加大民生投入，民生支出占公共财政支出76%。全面推进精准扶贫、精准脱贫，实现贫困人口预脱贫2.2万人。潮阳、潮南区被认定为海陆丰革命老区，纳入革命老区政策实施范围。新增城镇就业5.3万人、劳动力技能晋升培训2.56万人，城镇登记失业率2.46%。教育公平稳步推进，教育质量持续提升，学生综合素质全面提高，高考成绩稳居全省前列。7个区县全部通过“广东省推进教育现代化先进区”督导验收。公办义务教育、民办义务教育标准化学校覆盖率分别达100%和79.8%，公办及普惠性民办幼儿园、规范化幼儿园覆盖率分别达82%、71%。汕头大学、广东以色列理工学院列入广东省高水平大学重点学科建设高校，各有四个学科入选重点建设学科。全面推进中小学教师“县管校聘”改革、高中阶段学校考试招生制度改革，在全省率先建立乡村小学全科教师培养制度。区域医疗高地建设成效显著，汕大第一附属医院入选广东首批高水平医院建设“登峰计划”，眼科、护理、胸外科、烧伤外科进入全国百强行列，新建4个医学院士专家工作站，国家呼吸系统疾病临床医学研究中心在我市设立分中心，12家区县级医院和8家乡镇卫生院升级改造全面铺开。文化建设和文艺创作蓬勃发展，基层综合性文化服务中心建设基本实现全覆盖，成功举办“文化和自然遗产日”省主会场系列活动、群众艺术节等重大惠民文化活动。新增10个省级非遗项目，红色交通站陈列馆、蔡楚生电影史迹馆建成开放，樟林古港南粤古驿道保育活化工程基本建成，市非遗保护中心被评为全国非遗保护工作先进集体，潮阳区获评中国地名文化遗产“千年古县”。全民健身和竞技体育全面发展，新增社区（村居）健身场地186个、体育设施面积31万平方米，成功举办国际马拉松赛、“潮人杯”国际帆船赛、全国跳水冠军赛、全国帆板锦标赛等重大赛事。强化社保扩面征缴，参加城乡居民基本养老保险208.9万人、基本医疗保险501.6万人。加大社会救助力度，低保和医疗救助标准得到提升。老龄工作和养老事业稳步推进，银龄安康行动实现政府统保全覆盖。市慈爱托养中心、慈爱康复中心建成。公交服务提档升级，投放新能源公交车600辆，新增公交线路20条、优化13条，公交站点500米半径覆盖率达到95%以上。中心城区首批8个智能井筒式地下公共停车库开工建设，首个已建成投入使用。协商开放社会停车场，科学增设人行步道临时停车泊位。全面推进“平安汕头”建设，刑事警情、治安警情、交通事故死亡人数分别下降11.3%、14.1%、1.9%。连续8年未发生重特大安全生产事故。未发生系统性、区域性食品药品安全事故。深入开展扫黑除恶专项斗争。基层社会组织联合会建设的“汕头经验”在全国推广。全市党政军民众志成城，战胜“8·30”特大暴雨洪灾。2018年确定的十件民生实事完成年度任务。

一年来，我们不断树牢“四个意识”，坚定“四个自信”，坚决做到“两个维护”，把党的领导贯彻和体现到政府工作各个领域，政府自身建设全面加强。推进廉洁政府建设，落实政府部门党组主体责任，加强审计结果运用，强化廉政风险防控。继续开展“作风建设年”活动，认真落实中央八项规定精神，从严从实整治“四风”突出问题。推进法治政府建设，自觉接受人大、政协监督，办理人大议案、建议93件，政协提案245件，提请市人大常委会审议法规草案5件，制定政府规章9件。办理行政复议案件360件。国防动员、民族、宗教、港澳事务、对台、外事、侨务、残联、应急管理、信访、仲裁、气象、人防、地震、统计、档案、科协、新闻出版、社科、地方志等各项工作取得新进展。

各位代表！过去一年，我们所取得的成绩和进步，是习近平新时代中国特色社会主义思想科学指引的结果，是省委、省政府和市委正确领导的结果，是全市人民同心协力、接续奋斗的结果，是海内外乡亲和各界朋友大力支持、积极参与的结果。在此，我代表市人民政府，向全市广大工人、农民、知识分子、干部职工，向给予我们支持的人大代表、政协委员、各民主党派、工商联、社会各界人士，向中直和省直驻汕单位，向驻汕解放军、武警官兵，向所有关心和支持汕头发展的港澳台同胞、海外侨胞和国际友人，表示崇高的敬意和衷心的感谢！

当然，我们也清醒认识到，与人民群众、海内外潮人的期盼相比，特别是面对国际国内各种挑战和不确定因素，对照新时代新使命新定位，我市经济社会发展还存在很多亟待解决的问题：一是新旧动能转换有待加快，传统产业转型升级任务重，新产业新经济尚未形成规模，实体经济发展面临不少困难。二是科技创新能力不足，基础研究短板突出，高端人才缺乏，科研成果转化的创新体系尚未完善。三是农村投入欠账较多，道路、污水和垃圾处理设施等投入不足，环境脏乱差不同程度存在，部分地区水污染治理任重道远。四是基本公共服务质量和水平不高，公办优质学位相对不足，基层医疗服务能力有待提升，中心城区交通拥堵、停车难问题突出，与群众的期待还有不小差距。五是政府职能转变仍不到位，庸政懒政怠政现象依然存在，部分公职人员的担当意识、服务能力不强，群众和企业办事难问题还没有根本解决，一些领域不正之风和腐败问题仍然多发。对此，我们必须采取更加有力措施，切实加以解决。

二、2019年工作安排

今年是新中国成立70周年，是全面建成小康社会关键之年，是汕头的“落实发展年”。经过多年的发展和积累，特别是通过近年来的夯基础、补短板、强弱项，一大批事关当前与长远发展的重大项目和重大平台加速推进，一些制约汕头发展的短板正在逐步补齐。站在新的更高起点上，中央和省对汕头发展寄予厚望，习近平总书记视察广东时殷切嘱托，要加快汕头经济特区发展，把汕头作为重要发展极，打造现代化沿海经济带；省委十二届六次全会明确提出，要加快形成“一核一带一区”区域发展新格局，研究制定专门配套政策，支持汕头加快建设省域副中心城市，建设好华侨试验区、江湾新区等城市发展新平台。尽管外部环境复杂严峻，特别是受中美贸易摩擦影响，经济发展面临着许多不确定性。但只要我们紧扣重要战略机遇新内涵，加快经济结构优化升级，变压力为加快推动经济高质量发展的动力，拿出新时代改革开放再出发的汕头魄力，坚定新一轮发展的汕头自信，进一步认识自我、提升自我、超越自我，更加自觉地落实中央、省和市委的决策部署，更加主动地谋划和推动改革发展，就一定能够再创汕头新辉煌。

今年政府工作的总体要求是：高举习近平新时代中国特色社会主义思想伟大旗帜，全面贯彻党的十九大和十九届二中、三中全会以及中央经济工作会议精神，全面贯彻落实习近平总书记视察广东重要讲话精神，围绕落实“加快汕头经济特区发展，打造现代化沿海经济带重要发展极”的重大任务，以及省委十二届六次全会和市委十一届六次、八次全会的部署要求，坚持稳中求进工作总基调，坚持新发展理念，坚持推动高质量发展，坚持以供给侧结构性改革为主线，坚持深化市场化改革、扩大高水平开放，深入实施“三四四”发展思路，加快建设现代化经济体系，保持经济持续健康发展和社会大局稳定，加快建设省域副中心城市，打造活力特区、和美侨乡、粤东明珠。

今年经济社会发展的预期目标是：地区生产总值增长7%左右，固定资产投资额增长12%，社会消费品零售总额增长8.5%，外贸进出口总额增长1%，一般公共预算收入增长6%，居民消费价格指数涨幅控制在3%以内，常住居民人均可支配收入增长与经济增长基本同步，城镇登记失业率控制在3%以内，节能减排完成省下达的控制目标。

按照上述目标任务，重点抓好以下工作：

（一）用好改革开放关键一招，擦亮新时代经济特区牌子

推动特区再出发，必须高举新时代改革开放旗帜，在更高起点、更高层次、更高目标上推进改革开放，把特区的金字招牌擦得更亮。

深化营商环境综合改革。落实打造法治化国际化便利化营商环境三年行动计划，着力在开办企业、办理建设许可、获得电力、跨境贸易等重点领域争取更大突破，进一步减环节、压时限、降成本，增强企业办事满意率和获得感。深化综合行政执法改革，推动建立规范阳光执法体系。深化“数字政府”改革建设，加快“互联网+政务服务”平台建设，实现“一网通办”，力争企业与群众“只进一扇门，最多跑一次”。切实营造宜居宜业宜商宜创的法治化制度环境，依法保护民营企业和民营企业家合法权益，弘扬新时代企业家精神。加强经常性、规范化政企沟通，构建亲清新型政商关系。

加快重点领域改革。高质量完成市县机构改革任务，优化政府机构设置和职能配置，形成职责明确、运行高效的政府治理体系。加快推进事业单位改革，完成承担行政职能事业单位改革和从事生产经营活动事业单位转企改制任务。进一步推动国企改革重组发展，聚焦主业主责，开展混合所有制经济试点工作，逐步妥善解决历史遗留问题，推动国有资本做强做优做大。加快推进汕特联社改革重组，完成澄海、潮阳联社改制农商行工作。推进预算管理制度改革，完善财政资金分配、使用和管理制度。创新投资促进工作机制。深化投融资体制改革，全面实施市场准入负面清单制度，完善承诺制准入等管理方式，强化落实告知性备案、准入信息公示等配套措施。创新公共服务领域市场开放机制，制定社会资本进入垄断行业和特许经营领域实施办法。

推动全方位对外开放。深度参与国家“一带一路”建设，全面融入粤港澳大湾区，积极复制推广自贸试验区改革创新经验，在跨境贸易、投融资、财税政策、金融创新、出入境等方面探索创新政策体系和管理体制，建立与自由贸易相适应的体制机制。推进以“单一窗口”为核心的口岸信息化建设，继续压缩整体通关时间，规范口岸经营性收费，持续提升跨境贸易便利化水平。推动外贸稳中提质，出台外贸扶持政策，稳出口、扩进口、提质量，巩固传统市场、拓展新兴市场，培育发展跨境电商、市场采购、外贸综合服务等外贸新业态新模式，实现货物贸易和服务贸易双轮驱动。积极培育进口龙头企业，谋划建设集跨境电商、保税仓储、进口商品展示、国际供应链为一体的进口商品展示展销中心，配套完善口岸服务功能，争取国家批准设立冰鲜水产品、活牛等进境指定口岸，扩大大宗商品和优质消费品进口。出台进一步扩大对外开放积极利用外资若干措施，引导外资企业增资扩产。发挥南澳国家对台交流基地作用，加强对台交流与对台贸易工作。

（二）倾力打造“四大平台”，构筑大城市发展新格局

落实广东省《关于构建“一核一带一区”区域发展新格局促进全省区域协调发展的意见》，围绕现代化沿海经济带“串珠成链”空间布局，全面实施以功能区为引领的区域协调发展新战略，强化中心城区城市服务功能，统筹推进东部大发展、西部大开发、南部大建设、北部大提升，拉开大城市发展架构。

扎实推进“四大平台”建设。江湾新区要进入实质性规划建设。实施发展总体规划，加快编制江湾新区空间规划和各类专项规划，围绕构建“一带、两协同、三圈层、四组团”空间结构，启动24平方公里起步区建设，着力打造生态型科技新区；加快推进滨江大道规划建设，与汕湛高速、潮汕环线高速、牛田洋快速互联互通，形成新区骨架交通网；加快西胪、金灶、关埠、河溪四镇复合型公共服务中心和起步区特色服务中心的规划建设；加快推进产业平台搭建，建设蓝色新材料科技园、创新制造科技园、特色农产品电商物流园和现代综合物流园。华侨试验区要着力建设集经济、文化、教育、人才、金融于一体的高端示范区。精准对接华侨华人资源，发挥国家双创示范基地、“侨梦苑”和“中欧区域政策合作试点地区”等政策优势，着力构建全方位对外交流合作平台；规划建设华侨华人金融集聚区，筹建华侨证券公司；高水平规划建设大学园区，打造国家级孵化器基地；建设华侨大数据中心。自贸试验区要依托保税区、广澳港区加快申报工作。推进保税区扩区，围绕打造高端制造、研发设计、物流分拨、检测维修、销售服务等“五大中心”，谋划推进保税区转型升级为综合保税区。深入实施区港联动、港城融合，逐步将珠池港区的国际物流业务转移至广澳港区，推进广澳物流园、粤电LNG接收站等一批临港产业项目建设，大力发展海洋先进装备制造、新一代信息技术等现代化临港产业，做大做优临港经济。国家高新区要加快编制总体发展规划，推进中以（汕头）科技创新合作区建设，科学布局“科学园+孵化园+产业园+配套区”功能，推进园区基础设施建设和招商引智；出台支持科技创新政策，扶持孵化器和新型研发机构建设，完善全链条孵化育成和风险投资体系，吸引人才、技术、机构和项目集聚，建设集科研、教育、孵化、创新创业、特色小镇为一体的粤东科学城。

打造高水平全国性综合交通枢纽。着力提升汕头与粤港澳大湾区互联互通水平，推进实施一批重大交通基础设施工程。以厦深联络线通车为契机，增强以汕头火车站综合客运枢纽、广澳港区综合货运枢纽为核心的铁路、港口枢纽功能集成，推进汕潮揭区域综合交通共享互通，全力构建“承湾启西、北联腹地”的全国性枢纽节点。加快港口建设，广澳港区二期工程6月底前建成试运营，全面完成防波堤建设，加快推进三期工程前期工作；完善港口发展扶持政策，扩大班轮航线覆盖范围，加大航线密度。以提升与珠三角地区的通达水平为主攻方向，全面铺开建设汕汕高铁，打通“广汕汕+厦深”沿海高铁双通道；力争开工建设疏港铁路，推进粤东城际轨道网建设；汕湛高速建成通车，全面加快潮汕环线建设，启动建设南澳高速联络线，加快广东滨海旅游公路汕头段、汕南大道前期工作。加快推进城乡交通一体化，升级改造一批国省道和市政道路，加快汕头海湾隧道、牛田洋快速通道、凤东路和中山路东延、金砂西路西延、海滨路东延一期工程等建设，加快黄河路、泰山路快速化改造；启动潮汕大桥建设，推进跨汕头湾新通道、中心城区北部绕城快速通道和护堤路、金砂中路升级改造等前期工作。

加速城市扩容提质。启动新一轮城市空间规划编制，统筹推进“多规合一”。加快中心城区“一湾两岸”滨海城市空间形态塑造，有序推进东海岸新城、珠港新城、南滨新城建设，以“绣花”功夫保育活化小公园历史文化街区，推进开埠区修复二期工程，优化提升礐石风景区、桑浦山风景区，推动体育公园、北郊公园和沿梅溪河、新津河自然人文景观工程建设，增加公共开放空间，提升中心城区环境品质。持续推进城市更新，完成珠池路、练江路、珠峰路、东厦路、金湖路等一批道路升级改造，推进嵩山路、衡山路改造，继续推进第二批市政道路加铺沥青罩面，填补市政路网短板；配套完善城市地下管网，推进雨污分流，提升城市抗洪防涝能力；加快老旧小区微改造和乌桥岛棚户区、城中村改造。发挥中心城区引领作用，东融澄海、南澳，西联潮阳、潮南，加快城市化进程，促进全市一体化发展。进一步提升对汕潮揭城市群的牵引带动能力，加快资源共享、交通互通、产业互动，不断向着同城

（三）培育壮大现代产业，夯实高质量发展基础

坚持以供给侧结构性改革为主线，在“巩固、增强、提升、畅通”八字方针上下功夫，聚焦大项目、大园区、大产业，突出建链、补链、强链，打造高水平产业集群，发展贸、工、商一体化产业体系，构建高质量沿海产业带。

大力扶持民营经济发展。贯彻落实习近平总书记在民营企业座谈会上的重要讲话精神，坚持“两个毫不动摇”，坚持市领导走访企业制度，打造惠企暖企护企“汕头品牌”，切实解决民营企业的痛点、难点、堵点。落实国家新一轮更大规模减税降费政策，持续打好降低用地、用工、融资、社保、物流成本等“组合拳”。发挥国家产融合作试点城市优势，完善多层次资本市场，加强政银企合作，加大金融服务实体经济力度。推动资源要素向优势产业、优质企业集聚，重点支持上市企业、行业龙头企业、高成长企业，培育一批营收超100亿元、300亿元的龙头企业。发挥纾困基金作用稳定上市企业发展，继续支持有条件企业上市。支持大企业构建开放式“双创”平台，通过产业纽带、上下游配套、分工协作和技术扩散，促进大中小微企业协同发展。大力实施军民融合发展战略，推动军工技术与制造业有机融合，争取布局一批重大军民融合产业项目。

推动制造业高质量发展。深入实施新兴产业倍增计划，重点发展现代化临海产业，紧盯新一代信息技术、高端装备制造、海洋生物医药、新材料、新能源等比较优势新兴产业，推动超100亿元重大产业项目布局落地，培育发展一批产值超500亿元、超1000亿元产业集群。加快上海电气风电智能制造、比亚迪二期3D玻璃项目建设，吸引关联企业集聚，培育先进制造业基地。加快近海浅水区三峡、华能、大唐等5个风电项目建设，带动海上风电上下游产业发展。深化两化融合，选择优势产业领域若干骨干企业实施数字化、网络化、智能化改造提升示范试点，加快建设广东省智能化超声成像技术创新中心、广东省工业互联网创新应用试点区。加快传统优势产业改造提升，支持中小企业设备更新和骨干企业扩产增效，技改投资增长8%以上。全力推进“全国质量强市示范城市”创建工作，深入开展质量提升行动和对标达标专项行动，构建与产业发展相配套的公共检测技术服务体系。

做强做优现代服务业。大力发展现代金融业，实施“引金入汕”，积极引入银行、证券、保险、期货和投资性公司在我市设立区域总部或分支机构，推动城商行、农合机构、村镇银行加快发展，做大做强地方银行金融机构，增强区域金融中心功能。大力发展互联网、大数据、云计算等现代信息服务业，加快与信息产业龙头企业合作，依托汕头国际海缆登陆站，探索建设“离岸数据特区”。大力发展现代会展业，依托传统优势产业发展特色展会，培育具有国际采购功能的会展品牌，强化粤东地区会展业的龙头地位。大力发展全域旅游，创建全域旅游示范市，加快南澳国际旅游岛、小公园开埠历史文化旅游区、澄海前美侨文化风情小镇、南山湾国家级滨海旅游度假区等建设，发展滨海旅游、乡村旅游、红色旅游、美食旅游，培育规范特色民宿等旅游新业态，打造特色旅游品牌。大力开拓国内市场，深化与黑龙江鹤岗市对口合作，推动与鹤岗市互设特产体验中心、原产地中心仓库，促进南北货双向流通。推动电商快递基础设施合理化布局，提高电商与物流协同发展水平。充分发挥市供销系统流通服务网络优势，完善农产品冷链物流配送体系，推进“龙湖区智慧冷链物流”项目建设。加强市级重要商品储备管理，推动市场监测与应急商品数据库建设，加快肉类蔬菜流通追溯体系建设，提升市场应急管理水平。

促进产业园区提质增效。实施工业园区专项规划，规划建设澄海六合现代产城融合示范区，推动金平、龙湖区加快产业园区提质增效，加快潮阳、潮南区传统产业入园升级做强做大。明确园区主导产业，集聚引进产业链龙头企业和补链型企业，推进建设上海电气产业园区，打造具有特色优势的专业园区。创新园区发展模式，推广以租代供，用足用好低效产业用地再利用政策，通过限期开发、续期使用、产权分割、土地置换和协议收购等方式，开发建设一批园区通用厂房。完善产业园区综合配套，适当赋予经济事务审批权限。强化园区工作考核机制。

（四）深入实施创新驱动发展战略，打造区域科教文卫中心

坚持创新是第一动力，大力提升科技、教育、医疗、文化发展水平，全面提升省域副中心城市的虹吸力和辐射力。

着力构筑区域科创中心。制订出台加强基础与应用基础研究、促进科技创新高质量发展等政策措施，实施应用基础重大专项，建立稳定支持机制，全力推进重大科技基础设施和创新平台建设，着力提升我市原始创新能力，探索建立具有汕头特色的科创新模式。以新理念、新模式、新化工、新产业为目标，按照国内领先、国际有影响的标准，加快建设化学与精细化工广东省实验室，推进一期实验大楼、中试基地等基础设施建设，紧贴科学前沿，引进一流团队，做成一流平台，形成一流管理，建立以科研为根本、以首席科学家为核心、以课题为主导的体制机制，形成开放性、互动型的重大创新平台。鼓励引进名校名院在汕设立创新研究院，支持龙头企业、投资机构整合科研院所、高等院校力量，建立一批创新联合体、产业技术研究院和新型研发机构。实施高新技术企业培育提质工程，认定国家级高新技术企业200家以上，鼓励企业开展协同创新，促进科技成果转移转化。加大对具有自主核心技术中小企业的支持力度，充分发挥粤科汕头基金等政府引导性创业投资基金作用，加大对科技创业风险投资和创新孵化载体的投入引导，扶持科创企业登陆“科创板”，推动科技孵化育成体系提质增效。深化科技体制改革，推进科研项目和经费管理改革，围绕产业关键核心技术需求，探索实施定向组织、并行支持、悬赏揭榜等新型科研组织模式，带动企业和社会增加研发投入，力争R&D经费投入增长20%以上。强化知识产权创造、运用和保护，高标准建设国家知识产权示范城市。全面落实加快人才发展实施意见，精准对接高端人才服务需求，形成人才政策叠加效应。

着力构筑区域教育高地。加快发展学前教育和高等教育，巩固提升基础教育，全力推进创建教育现代化先进市。以普惠性学前教育为重点，新建一批公办幼儿园，支持农村幼儿园建设，公办园和普惠性民办园在园幼儿占比达到80%。推动高等教育扩规模、提质量，支持汕头大学建设“广东省高等教育体制机制改革实验示范校”，加快广东以色列理工学院南校区建设；积极引进国内外高水平大学设立校区或合作办学；启动广东汕头幼儿师范高等专科学校建设，支持汕头技师学院扩建，加快粤东技师学院、汕头技师学院纳入高等职业教育序列，汕头卫生健康学院招生办学。推进义务教育均衡发展，统筹推进县域内城乡义务教育一体化，确保城镇充足学位供给。推动高中教育优质特色发展，深入推进高中阶段学校考试招生制度改革试点，扩大普通高中自主招生试点。提升中等职业教育水平，鼓励优质中职学校通过兼并、托管、合作办学等方式整合办学资源，加快推进市属中职教育资源的整合优化。加强教师队伍建设，全面落实中小学教师“县管校聘”管理制度。积极开展中小学生校外托管机构治理和校内课后服务。认真做好市县级政府履行教育职责考评。

着力构筑区域医疗高地。实施构建区域医疗高地“十百千工程”。支持汕大第一附属医院高水平医院建设“登峰计划”，加快第一附属医院、澄海区人民医院、潮阳区中医院、潮南区人民医院异地新建和国瑞医院、宜华医院建设，力争新增2家三甲医院。逐级打造百个临床重点专科，努力申报省级临床医学研究中心，争取一批院士专家工作站和国家医学创新平台落户汕头。分批次培养引进千名高端医学人才。全力推进区域医疗中心项目建设，加强基层医疗能力建设，加大力度推动区县级医院能力提升和乡镇卫生院升级改造，建成一批村卫生站。加强以市中心医院、汕大第一附属医院等为牵头单位的医联体建设，带动基层医疗卫生机构达标建设。加强基层医疗卫生人才培训，为每万名城乡居民配备3名合格全科医生。加强医德医风建设，纠正医药购销领域和医疗服务中不正之风，构建和谐医患关系。

着力构筑区域文化高地。拓展优化城市历史文化游径，规划建设一批集文化展示、旅游观光等功能于一体的高水平文化场馆。完善市级重点公共文化设施和基层综合性文化服务中心配套建设，打造城市文化地标。完成汕头城市发展展览馆建设，推进小公园专题博物馆群建设，建成市档案馆新馆，创建粤东文物考古工作中心。深入挖掘优秀潮汕文化和侨文化，大力宣传蔡楚生、郑正秋、陈波儿等潮籍电影先驱事迹，讲好汕头故事。加大非遗保护传承与创新力度，拓展对外文化交流渠道，繁荣文化艺术精品创作，办好汕头潮剧节、群众艺术节等重大活动，举办高雅艺术系列惠民演出。加快文化产业园区、版权兴业示范基地建设，做大做强文化创意等现代文化产业。深入开展“扫黄打非”工作，净化文化市场环境。全面推进体育强市建设，广泛开展全民健身活动，办好2019汕头国际马拉松赛等大型赛事，新增社区健身场地90个、体育设施面积10万平方米，打造15分钟健身圈。

（五）大力实施乡村振兴战略，不断提高城乡发展的平衡性和协调性

落实乡村振兴战略实施意见，按照“清垃圾、治污水、修村道、兴产业、强组织”要求，集中资源力量解决农村短板弱项，加快农村城市化进程，推动城乡融合发展。

坚决打好精准脱贫攻坚战。持续推进就业扶贫、技能扶贫，多渠道开发岗位帮扶贫困户就地就近就业。大力发展产业扶贫，扶持发展农村二三产业，规范发展资产收益扶贫项目。加大临时救助力度，及时将符合条件的返贫人口纳入救助范围。深入开展扶贫领域腐败和作风问题专项治理，确保脱贫攻坚取得明显成效。

大力推动乡村产业振兴。深化农业供给侧结构性改革，稳步推进农村集体产权制度改革，完善农村集体“三资”管理平台建设。实施新型乡村助农服务示范体系“百千工程”，推进电商进农村示范县、农村电商驿站、特色小镇建设。着力发展现代高效农业，培育壮大新型农业经营主体，推进现代农业产业园建设。大力发展“一村一品、一镇一业”，实施富民兴村特色产业培育工程，鼓励发展农产品加工、休闲农业与乡村旅游，促进农村一二三产业融合发展。

全域推进美丽乡村建设。完善“村收集、镇转运、区处理”的农村生活垃圾处理机制，完成45座镇级生活垃圾填埋场的整治验收。加快推进以PPP模式整区（县）建设村镇污水处理设施。加快“四好农村路”建设，建设安保工程230公里、“三通工程”163公里，改造危桥30座。推进潮南区5个省级新农村连片示范工程项目建设。积极盘活利用农村闲散土地资源，鼓励危旧空置房改造盘活，支持农民公寓建设，探索休闲农业、乡村旅游项目点状供地模式，提高节约集约用地水平。推进旧村庄改造，加强农村基础设施、社会事业建设，建成富有潮汕特色的美丽宜居村（社区）238个。

（六）坚决打好污染防治攻坚战，建设绿水青山美丽汕头

牢固树立绿水青山就是金山银山的发展理念，全面落实河长制湖长制，加快补齐生态环境短板，全力打好碧水攻坚战、蓝天保卫战和净土防御战，推动生态文明建设迈上新台阶。

坚决打赢练江流域综合整治攻坚战。集中人力财力物力加快环保基础设施建设，流域内污水处理设施及配套管网年内建成并投入运营，启动潮阳、潮南区生活垃圾焚烧发电厂二期工程建设，如期建成潮阳、潮南区纺织印染环保综合处理中心。综合施策系统治理，持续开展五大专项行动，全面减少工业、农业和生活污染源，加快推进河涌整治、水利工程建设、堤岸绿化美化，提升干流纳洪、行洪能力，确保练江流域重点支流基本消除黑臭，干流水质明显好转。

强化污染治理和生态建设。全面打好污染防治攻坚战。加快推进中小河流治理和三江水系连通工程。完成县级饮用水源保护区环境问题清理整治工作，落实饮用水水源地规范化建设。加大力度保护韩江水质，推进引韩供水工程，保障供水安全。推进创建节水型城市，逐步实现节水减排。深入实施水体达标方案，完成全市13宗城市建成区黑臭水体整治，加快推进金平、龙湖区其它污染水体综合治理，确保梅溪河升平断面稳定达标。推进中心城区西片区污水处理设施及配套管网建设，启动龙珠水质净化厂迁建。加快集中供热系统建设。建成机动车排气污染遥感监测系统。加快危险废物处理中心和雷打石环保电厂二期建设。积极推进城乡生活垃圾分类工作。编制实施生态环境“三线一单”，建立生态环境分区管控体系。大力推广绿色建筑和建筑节能。加强林业生态建设，重点管护好牛田洋红树林景观带。加快平屿西国家级海洋牧场、青澳湾国家级海洋公园建设，推进汕头内海湾整治修复，守护好沿海美丽自然生态。

（七）增强民生福祉，形成共建共治共享社会治理格局

坚持以人民为中心的发展思想，不断提高基本公共服务水平和质量，不断提升社会治理智能化、科学化、精准化水平，让群众有更多获得感、幸福感、安全感。

全力推进创文提质升级。深化城市环境综合整治，深入开展以拆除违章建筑为重点的专项整治，继续推动农贸市场升级改造等。实现城市建成区数字城管全覆盖，完善城市管理考核奖罚机制。推进城市绿化彩化美化亮化，加快七日红公园、华侨公园、石炮台公园、金砂公园及中心城区绿化平台改造升级，提升中心城区景观照明水平。深入推进“厕所革命”，新建、改建公厕300座以上。深入开展公共交通行业服务质量提升年活动，推进公交路权优先，完善公交线网布局，加快公交站场建设，不断提升公交出行分担率。推进巡游、网约出租车融合发展，投放1000辆新能源出租车。优化内海湾轮渡码头功能，完善客轮线路规划，加密轮渡班次，发展水上公交。建成首批8个井筒式地下公共停车库，继续科学规划，综合利用边角公共用地，通过城市微改造，加快推广建设地下公共停车库；在市区道路、人行道科学设置临时停车泊位，新增停车位1万个，建成全市停车管理服务平台，实现智能化高效管理。深化文明交通提质升级，开展“雷闪”“飞鹰”“清患”系列交通秩序专项整治行动，加大路面执法力度，规范学校、医院、市场等重点公共场所交通秩序，建设一批人行天桥，推动交通秩序大改观。广泛开展社会风尚提升行动，深入推进文明村镇、文明单位、文明家庭、文明校园等群众性创建活动，壮大向上向善的社会正能量。

加强和创新社会治理。打好防范化解重大风险攻坚战，加强地方金融监管和风险预警，坚决防范化解地方政府隐性债务风险，坚决制止违法违规举债。推进国家社会信用体系建设示范城市创建工作，提升全社会诚信水平。强化应急管理，提高突发事件防范处置和防灾救灾减灾能力。严格落实安全生产责任制，坚决遏制重特大事故发生。深入开展食品药品安全专项整治，严厉打击制假售假违法行为。落实粮食安全政府责任制，完善粮食安全保障体系。持续开展扫黑除恶专项斗争，铲除黑恶势力滋生土壤。强化社会治安综合治理，深入实施公安“大数据”和“智慧新警务”战略，规划建设反恐维稳指挥中心、智慧视频平台，完善智能交通体系，提升社会治安防控智能化、科学化、精准化水平。推动全民禁毒工程，完善反恐责任体系，严厉打击违法犯罪活动。加强和改进信访维稳，积极防范化解社会矛盾。创新基层社会治理，实施“双百镇（街）社会工作服务五年计划”，充分发挥社工组织作用。加强社区管理服务，推动住宅小区业主自治。

切实保障和改善民生。实施就业优先战略和积极就业政策，发挥省市共建创业孵化基地带动就业功能，新增城镇就业4.8万人、劳动力技能晋升培训2.58万人。完善职工工资正常增长机制，拓宽居民劳动收入和财产性收入渠道，进一步缩小城乡居民收入差距。完善社会保障体系，扎实推进企业职工养老保险省级统筹工作基金，探索被征地农民以灵活就业人员身份参加企业职工养老保险。深入推进医保制度改革，稳妥推进医保城乡一体化改革试点，全面实施按病种分值付费，开展村卫生站纳入医保支付试点工作。加大底线民生保障力度，提高城乡低保、特困人员救助供养标准，完善医疗救助一站式结算系统。推进养老服务体系建设，加快推进市社会福利中心、市救助安置中心建设。做好国防动员、双拥优抚、退役军人服务管理保障工作，加强国防教育基地建设，巩固军政军民团结。

继续办好十件民生实事。计划投入资金25.15亿元，集中力量办好十件民生实事。一是加大教育投入力度。二是强化底线民生保障。三是提高城乡居民养老保障水平。四是保障城乡居民福利。五是提升城乡卫生医疗服务能力。六是实施城乡环境卫生治理。七是改善农村生产生活条件。八是加大环保整治力度。九是实施交通便民工程。十是提升文化惠民水平。

（八）加强政府自身建设，打造人民满意政府

坚决落实全面从严治党要求，深入推进政府职能转变，转作风、强担当、重落实，不断提升政府公信力和执行力，推动政府工作在新的一年有新气象新作为。

加强党的领导和党的建设。把党的政治建设摆在首位，树牢“四个意识”，坚定“四个自信”，坚决做到“两个维护”，自觉在思想上政治上行动上同党中央保持高度一致。严守政治纪律和政治规矩，严肃党内政治生活，严格执行新形势下党内政治生活若干准则。

加强法治政府建设。严格遵守宪法和法律，善于运用法治思维和法治方式开展工作。自觉接受人大法律监督、工作监督和政协民主监督，认真听取各民主党派、工商联、无党派人士和人民团体的意见，办好各类建议和提案。坚持科学民主依法决策，推行行政程序法定化，强化行政权力监督，推动政府工作全面纳入法治轨道。加强政府立法工作，认真组织实施立法计划，引导社会各方有序参与立法。严格规范公正文明执法，全面落实行政执法责任制。加大全民普法力度，深入开展“七五普法”。依法加强经济管理，全力做好第四次全国经济普查。

坚持廉洁从政。强化主体责任，落实“一岗双责”，以永远在路上的执着推进廉洁政府建设。严格落实中央八项规定及其实施细则，坚持不懈纠“四风”，紧盯形式主义、官僚主义新动向新表现，严厉整治群众身边的不正之风和“微腐败”。改革审计管理体制，加大审计监督力度，严管公共资金和公共资源交易，严控“三公”经费支出。深化政务公开，保障人民群众的知情权和监督权，从源头防治腐败。加强干部队伍建设，完善激励约束、容错纠错机制，努力打造忠诚、干净、担当的高素质公务员队伍。

各位代表，使命开启未来，实干铸就梦想！让我们更加紧密地团结在以习近平同志为核心的党中央周围，高举习近平新时代中国特色社会主义思想伟大旗帜，在省委、省政府和市委的坚强领导下，闻鸡起舞、日夜兼程、风雨无阻，以新担当新作为推动特区再出发，奋力开创新时代汕头改革开放新局面，以优异成绩庆祝中华人民共和国成立70周年！
